# The Regulation of Inflammatory Mediators in Acute Kidney Injury via Exogenous Mesenchymal Stem Cells

**DOI:** 10.1155/2014/261697

**Published:** 2014-04-15

**Authors:** Tao Du, Ying-Jian Zhu

**Affiliations:** ^1^Department of Urology, School of Medicine, Shanghai First People's Hospital, Shanghai Jiao Tong University, No. 100 Haining Road, Shanghai 200080, China; ^2^Department of Urology, Henan Provincial People's Hospital, No. 7 Weiwu Road, Zhengzhou 450003, China

## Abstract

Acute kidney injury (AKI) remains to be an independent risk factor for mortality and morbidity. Inflammation is believed to play a major role in the pathophysiology of AKI. Exogenous mesenchymal stem cells (MSCs) are now under extensive investigation as a potential therapy for AKI. Various preclinical studies indicated the beneficial effects of MSCs in alleviating renal injury and accelerating tissue repair. However the mechanisms responsible for these effects are incompletely understood. In the recent years, anti-inflammatory/immunoregulatory properties of MSCs have become one of the important issues in the treatment of AKI. This review will summarize the current literature on the regulation of inflammatory mediators via exogenous MSCs contributing to the recovery from AKI.

## 1. Introduction

Acute kidney injury (AKI) is defined as a rapid decrease in glomerular filtration rate (GFR) caused by both vascular and tubular factors, including increased renal vasoconstriction, loss of autoregulation, and tubular obstruction [1–3]. Although incidence rates are decreasing, AKI is still associated with a high mortality rate [4]. Inflammation is now believed to play a major role in the pathophysiology of AKI [5, 6]. Endothelial injury can increase microvascular permeability which may lead to inflammatory cells recruitment into the injured kidney during the initial process of AKI [7]. Proximal tubular cells under hypoxia-induced damage can produce proinflammatory and profibrotic factors that result in infiltration of inflammatory cells into the injured kidney [8]. In the recent years, some anti-inflammatory therapies, such as lymphocyte or macrophage depletion, have been used against inflammatory targets for prevention and treatment of AKI [9]. Exogenous mesenchymal stem cells (MSCs) have been considered as one of the new effective strategies for AKI recently. Although the mechanisms responsible for their protective and regenerative effects are incompletely understood, anti-inflammatory/immunoregulatory properties of MSCs are recognized as one of the important mechanisms. The present brief review hopes to focus on the role of exogenous MSCs to ameliorate kidney injury and accelerate kidney repair in AKI through regulating inflammatory mediators.

## 2. Mesenchymal Stem Cells (MSCs)

Mesenchymal stem cells (MSCs), also known as mesenchymal stromal cells, have been the focus of great interest in regenerative medicine for their potential therapeutic applications in AKI. Since nephrons are largely of mesenchymal origin and stromal cells are of crucial importance for signaling leading to the differentiation of both nephrons and collecting ducts, MSCs are undifferentiated adult cells and may be isolated from bone marrow, umbilical cord, adipose tissue, placenta, synovium, and skeletal muscle [10–14]. They are characterized by three main criteria: (a) the ability to differentiate into osteoblasts, adipocytes, and chondroblasts* in vitro*; (b) the expression of surface markers CD73, CD90, and CD105; lack of expression of haematopoietic markers including CD34 and CD45; (c) plastic adherence in culture [15]. Recent studies suggested that administering exogenous MSCs could ameliorate renal injury and accelerate renal repair in AKI (Table 1). Exogenous MSCs appeared to make renoprotective effects by several mechanisms, including (a) engraftment and differentiation of MSCs into the host tissue or organ; (b) therapeutic fusion into one with existing host cells; (c) stimulation of endogenous repair by regenerating local resident SCs; and (d) release of paracrine and/or endocrine signals from MSCs. In the last decade, almost all the studies showed that the therapeutic effects of MSCs are mainly mediated through paracrine rather than differentiative mechanism. They have identified over forty growth factors, cytokines and chemokines secreted from MSCs, such as hepatocyte growth factor (HGF), insulin-like growth factor 1 (IGF-1), vascular endothelial growth factor (VEGF), interleukin-1 (IL-1), IL-4, IL-5, IL-6, KC, CXCL16, CCL2, CCL3, CX3CL1, and CCL5 [16, 17]. Some of them enhance cell proliferation, reduce cell apoptosis, modulate the inflammation, and are good candidates for therapeutic effects in AKI. Given the importance of inflammation in the pathophysiology of AKI, it is very imperative to discuss these anti-inflammatory/immunoregulatory properties of MSCs and the role they play in renoprotection [5].

## 3. Cytokines and Chemokines

Many cytokines and chemokines are released by leukocytes, renal tubular cells, mesangial cells, endothelial cells, and platelets into the injured tissue [29–33]. They are also important components for both the initiation and extension of inflammation. Exogenous MSCs would protect kidney from AKI by regulating the inflammatory-related cytokines and chemokines. Chemokine receptors may assist MSCs in migrating to sites of inflammation and participating in the regulation of inflammation.

### 3.1. Cytokines

Cisplatin-induced AKI is associated with increases in the cytokines IL-1*β*, IL-6, and IL-18 and neutrophil infiltration in the kidney [34]. The expression of tumor necrosis factor-*α* (TNF-*α*) is increased in cisplatin-induced AKI [35]. TNFR2 also participates in cisplatin-induced AKI and may play an important role in TNF-*α* mediated inflammation in kidney [36]. In the model of ischemic AKI, some proinflammatory cytokines are increased in kidney [37–39]. In response to the stimulation with noxious stimuli, endotoxin, and hypoxia in AKI, administered MSCs would reduce the expression of proinflammatory cytokines and increase the expression of anti-inflammatory factors in kidney. In the model with ischemic AKI, MSCs significantly reduced the expression of proinflammatory cytokines IL-1*β*, TNF-*α*, interferon-gamma (IFN-*γ*), and inducible nitric oxide synthase (iNOS) and highly upregulated the expression of anti-inflammatory cytokines IL-10, basic fibroblast growth factor (bFGF), TGF-*α*, and Bcl-2 in kidney [18]. In another study with ischemic AKI, the expression of IL-6 and TNF-*α* was significantly lower, while the expression of VEGF was significantly higher in kidney after MSCs treatment [40]. When cisplatin-treated mice were injected with MSCs, some cytokines in serum, such as MIP-2, IL-6, and IFN-*γ*, were significantly lowered and renal injury was ameliorated [19]. Several growth factors released from MSCs, such as VEGF, fibroblast growth factor-2 (FGF-2), HGF, and IGF-1, would improve blood flow, promote cell growth, decrease cell apoptosis, and exert a beneficial effect on neovascularization and tissue remodeling [16, 41–44]. Some studies also found that MSC-conditioned medium (MSC-CM) might protect renal cells from apoptosis induced by cisplatin* in vitro* [17, 45].

### 3.2. Chemokines

Chemokines are a family of chemotactic cytokines that were initially identified on the basis of their ability to induce the migration of different cell types [46]. A large number of proinflammatory chemokines, for example, CCL2, CCL5, CXCL8, and CXCL12, are upregulated in kidney after ischemic AKI, and chemokine receptor expressing inflammatory cells are attracted by these chemokines. These results lead to marked neutrophil infiltration in kidney [47, 48]. Some proinflammatory chemokines are controlled at the transcriptional level by nuclear factor-kB (NF-kB) and activating protein-1 (AP-1), which are activated by the phosphorylation of p38 MAPK. And pharmacological inhibition of p38 MAPK might significantly reduce proinflammatory chemokines production, attenuate leukocyte infiltration, and prevent tubular necrosis in a mouse model of ischemic AKI [49].

MSCs express several chemokines and chemokine receptors (Table 2). The chemokine receptors may assist in their migration to the sites of inflammation and participate in the regulation of inflammation. Some chemokine receptor genes, such as CXCR3, CXCR5, CCR1, CCR7, and CX3CR1, were upregulated in human bone marrow-mesenchymal stem cells (hBM-MSCs), while human umbilical cord Wharton's jelly-mesenchymal stem cells (hUCWJ-MSCs) showed higher expression for CCR3 [50]. Short-term exposure of MSCs to low oxygen increased the expression of chemokine receptors CX3CR1 and CXCR4 and enhanced their engraftment* in vivo* [51]. Hypoxic preconditioning mesenchymal stem cells (HP-MSCs) enhanced the expression of CXCR4 and CXCR7, which not only improved MSCs' chemotaxis but also stimulated the secretion of proangiogenic and mitogenic factors [52].

In response to the stimulation with noxious stimuli, endotoxin and hypoxia in AKI, administered MSCs would reduce the expression of proinflammatory chemokines and ameliorate kidney injury. MSCs could decrease the expression of MIP-2, CCL2, and KC in plasma and reduce the toxicity of cisplatin on kidney* in vivo *[19]. MSCs could decrease mRNA levels of CXCL1, CXCL2, CXCL5, CCL2, and CCL3 in kidney in sepsis-associated mice AKI and improve the recovery of tubular function [20]. In ischemic AKI, MSCs could also decrease the expression of CCL2, CCL3, CCL5, and KC in kidney and reduce acute tubular necrosis in injured kidney [21].

## 4. Adhesion Molecules

Adhesion molecules are required for leukocyte adhesion during inflammation. Leukocyte adhesion to endothelial cells leads to inflammation and extension of cellular injury [61]. Intercellular adhesion molecule-1 (ICAM-1) plays an important role in the pathophysiology of AKI [62]. The administration of a monoclonal ICAM-1 antibody or ICAM-1 deficient mice is protected against renal ischemia [63, 64]. In the model of kidney transplantation, MSCs could reduce the gene expression of proinflammatory cytokines/chemokines and ICAM-1 and ameliorate inflammation induced by prolonged cold ischemia [65]. In another study with ischemic AKI, kallikrein-modified mesenchymal stem cells (TK-MSCs) can provide enhanced protection against AKI by inhibiting apoptosis and inflammation. The expression of proinflammatory mediators CCL-2 and ICAM-1 was significantly reduced in the TK-MSCs group [66].

Selectins and their ligands are other important adhesion molecules that participate in the inflammatory response. P-selectin is expressed as part of the inflammatory stimulus in platelets and endothelial cells; L-selectin is expressed in leukocytes and lymphocytes; and E-selectin is expressed in endothelium. Renal ischemia has been shown to be associated with upregulation of endothelial P-selectin and enhanced adhesion of neutrophils [67].

Although there is no report about the expression of selectins in injured kidney after MSCs' treatment, P-selectin and its counter-ligand were found to be involved in the extravasation of MSCs [68]. Intravenously administered MSCs can roll along the walls of the blood vessels in the ear veins of mice, and this phenomenon was significantly decreased in mice genetically deficient of P-selectin. E-selectin and L-selectin have been reported to be absent or present only in low amounts on MSCs, and their significance in MSC trafficking, compared with P-selectin, may be unimportant.

## 5. Inflammatory Cells

Inflammatory cells play a central role in the pathogenesis of AKI. Exogenous MSCs showed a beneficial effect on ameliorating kidney injury and accelerating kidney repair. Administrated MSCs may decrease the infiltration of neutrophils and macrophages, inhibit the dendritic cells' differentiation and maturation, reduce the lymphocytes activities, and decline the cytotoxic activity of NK cells in injured kidney.

### 5.1. Neutrophils

Neutrophil recruitment is an important early step in controlling tissue infections or injury. Neutrophil chemoattractants CXCL1/CXCL2 produced by mast cells and macrophages initiate an early stage of neutrophil recruitment during tissue inflammation [69]. MSCs can decrease a panel of inflammatory cytokines and reduce the inflammatory infiltration of macrophages and neutrophils in kidney when following kidney injury [70]. The improvement of inflammatory responses in animal models was at least partially explained by the NF*κ*B-dependent secretion of soluble tumour necrosis factor receptor-1 (sTNFR1) by MSCs. In a mouse model of sepsis-associated AKI, MSCs would alleviate kidney injury and attenuate neutrophils' infiltration in kidney [20]. In another mouse model with cisplatin-induced AKI, infusion of MSCs ameliorated both renal function and tubular cell injury and prolonged survival. Transplanted MSCs might localize in peritubular areas and limit capillary alterations and neutrophils' infiltration [71]. Human MSCs, pretreatment with an antioxidant, might improve the efficacy of MSCs' transplantation [72]. Administration of these pretreated MSCs could decrease inflammation and fibrosis and reduce neutrophils' infiltration in injured tissue.

### 5.2. Lymphocytes

The role of lymphocytes in AKI is an ongoing area of study. Lymphocytes have been shown to be important modulators of innate and adaptive inflammatory responses in AKI models [73].

MSCs can regulate T cells function and make their therapeutic effects in AKI [74]. MSCs can inhibit T cells activities. They can inhibit the effector T cells both* in vitro* and* in vivo *[75, 76]. MSCs could also suppress AKI-induced T cells infiltrating in ischemic kidney. When cultured with T cells, MSCs either from the same donor or from different donor can induce a G0/G1 checkpoint arrest in T cells [77, 78]. The mechanisms by which MSCs are able to mediate immunosuppression of T cells are diverse and complex; several secreted effectors have been linked to this process. Among them, indoleamine 2,3-dioxygenase (IDO), prostaglandin E2 (PGE2), TGF-*β*, and HGF have been described to play major roles [79–81]. MSCs can also display potent immunosuppressive effects on lymphocyte responses. These effects included the prevention of lymphocyte activation as well as the suppression of T cells proliferation. These effects were mediated through the expression of COX1/COX2 enzymes and by the production of PGE2 [82]. Moreover, MSCs can stimulate the production of regulatory T cells (Tregs), leading to self-regulation of the immune response. Through interaction with splenocyte, MSCs may induce more Tregs and attenuate ischemic AKI. Coculture of splenocytes with MSCs could increase the number of Tregs* in vitro *[74]. Tregs are commonly identified by their expression of CD4 and CD25 on the cell surface and upregulation of the transcription factor FoxP3. MSCs could expand CD4+CD25highFoxp3+ regulatory T cells via HLA-G5 secretion in peripheral blood lymphocytes [83].

MSCs also have an important immunosuppressive action on suppression of B cells proliferation. Mice deficient in B cells are protected against ischemic AKI [84]. In a murine model of systemic lupus erythematosus, MSCs could inhibit the activation of B cells with IFN-*γ* [85]. MSCs could also suppress the activation of B cells via inhibiting the production of B cell-activating factor (BAFF) [86].

### 5.3. Natural Killer Cells

Natural killer (NK) cells are a type of lymphocytes that mediates innate immunity against pathogens and inflammation via their ability to secrete cytokines [87]. NK cells are important participants in the early-stage innate immune responses in ischemia AKI. A recent study demonstrated that NK cells could directly kill tubular epithelial cells. NK cells induced apoptosis in tubular epithelial cells and contributed to renal ischemia-reperfusion injury (IRI). NK cells depletion in wild-type mice was protective against AKI, while adoptive transfer of NK cells worsened kidney injury in NK cell, T cell, and B cell-null Rag2 (−/−) gamma(c)(−/−) mice with IRI [88]. MSCs could inhibit NK cells proliferation and cytotoxic activity. The inhibition was mediated by a soluble factor generated upon incubation with NK cells activated by IL-15 or IL-2 [89]. MSCs can inhibit NK cells function. Through human leukocyte antigen-G5 (HLA-G5), MSCs can affect innate immunity by inhibiting both NK cell-mediated cytolysis and IFN-*γ* secretion [90]. MSCs can hamper the NK cells-mediated immune response by preventing their acquisition of lymphoblast characteristics, activation, and changing the expression profile of proteins with an important role in immune function [91]. Moreover, MSCs may inhibit the cytolytic functions of NK cells and have negative effects on the NK-mediated graft-versus-leukemia (GVL) [92].

### 5.4. Macrophages

Macrophages are important effector cells in both the adaptive and the innate immune response. They can mediate inflammation in the interstitium of the outer medulla, which has been described early in the course of ischemic AKI. Although the precise mechanisms of macrophage-mediated renal injury have yet to be determined, it is evident that macrophages can produce many molecules to cause renal damage. Some proinflammatory factors, for example, TNF-*α*, IL-1, TGF-*β*1, and PDGF secreted by macrophages, are thought to play important roles in the pathogenesis of the disease [93]. Apart from tissue destruction, macrophages can also play roles in tissue homeostasis, cellular replacement, and tissue repair [94]. Macrophages can phagocytose and remove apoptotic cells, deposited immune complexes, and fibrin. In addition, macrophages can secrete HGF and VEGF, which can promote the repair of damaged tubules and endothelium.

Some researchers reported that AKI induced kidney inflammation and fibrosis involving M1 and M2 macrophages, respectively [95, 96]. M1 macrophages predominate in the early stage of AKI and produce multiple cytokines and chemokines to recruit the immune cells and activate the innate host defense. M2 macrophages include M2a, M2b, and M2c subtypes. M2 macrophages predominate in the late stage of AKI. They are alternatively activated according to the defined stimulation and promote angiogenesis and mediate wound healing, extracellular matrix (ECM) deposition, and tissue remodeling. With ongoing injury, sustained macrophage infiltration may result in the continuous production of various wound-healing growth factors and ultimately lead to irreversible fibrosis, tissue destruction, and progressive chronic kidney disease [97]. In the recent years, some researchers proposed an alternative view to classify macrophage phenotypes as proinflammatory, anti-inflammatory, profibrotic, and fibrolytic macrophages according to their different microenvironments and functional characteristics [98]. As the injury and repair process shifts from the initial inflammatory phase to that of remodeling phase, macrophages subsequently exhibit varying polarization states and exert a diverse range of functional activities.

Several studies have demonstrated that MSCs could inhibit the expression of macrophage in the initial period of AKI. In a mouse model with cisplatin-induced AKI, infusion of mesenchymal-like progenitors (MPs) derived from human embryonic stem cells (hESCs) reduced renal macrophage infiltration through regulating MCP-1 and promoted the recovery from AKI via paracrine mechanism [99]. In a rat model of ischemic AKI, intravenous administration of allogenic fetal membrane-derived mesenchymal stem cells (FM-MSCs) could significantly suppress macrophage infiltration and renal tubular apoptosis [22]. Recent studies have also demonstrated that, in ischemic AKI, anti-inflammatory macrophages were markedly increased in kidney in the late period when human umbilical cord-derived stromal cells (hUCSCs) were injected intravenously [100, 101].

### 5.5. Dendritic Cells

Dendritic cells (DCs) have a dual role and can induce tolerance or immunity. In the steady state, immature DCs (iDCs) situated in the renal interstitial microenvironment are affected by autophagy of proteins from dying cells, cell-to-cell contact, danger-associated molecular patterns (DAMPS), pathogen-associated molecular patterns (PAMPS), humoral mediators, or filtered antigens [102]. Without tissue damage or local inflammation, these iDCs express low amounts of costimulatory molecules. During an infection or in the presence of maturation-inducing inflammatory signals, mature DCs will induce the development of effector T-cell responses. DCs may play an important role on the production of cytokines and chemokines that drive neutrophils' infiltration in ischemic AKI.

MSCs are able to alter the cytokine production of DCs, resulting in a more tolerant and/or anti-inflammatory phenotype. MSCs have also been known to interact with DCs, making them become regulatory DCs [103–105]. In the mouse model of ischemic AKI, MSCs are partially mediated via DCs to induce the immune tolerance and play an important role in alleviating kidney injury [102]. MSCs, treated with 14S, 21R-dihydroxy-docosahexaenoic acid, could enhance the efficiency in improving the renal function, reducing renal tubular cell death, and inhibiting infiltration of neutrophils, macrophages, and DCs in ischemic AKI [106].* In vitro*, MSCs mediated a potent inhibition on DC differentiation, and the inhibition was restricted to the early stages of cytokine-induced progression from monocytes to iDCs. The inhibition affected both the expression of informative DC surface markers and the acquisition of DC functions, such as IL-12 production. The inhibitory effect is primarily mediated by PGE2 [107, 108].

## 6. Microvesicles Derived from MSCs in AKI

It has been suggested that MSCs can alleviate AKI by providing a paracrine support rather than replacing renal tubular cells to the repair. Further support for the paracrine action of MSCs is provided by the experiments showing that MSC-CM can mimic the beneficial effects as the cells of origin [16, 44, 45]. Microvesicles (MVs) from MSCs, as one of the components in the cell-to-cell communication network, are involved in tissue regeneration and therefore contribute to the paracrine action of MSCs [109, 110]. MVs are vesicles composed by exosomes, shedding vesicles, and apoptotic bodies. Exosomes range from 30 to 120 nm in size, and their release depends on cytoskeleton activation, while vesicles generated by direct budding of the plasma membrane, also known as shedding vesicles, are more heterogeneous in size and ranging from 100 nm to 1 *μ*m in size [111]. In our study, MVs derived from hUCWJ-MSCs were positive for some surface expressed molecules, such as CD9, CD44, CD63, and CD73, and negative for CD34 and CD45 [112]. MVs may influence the behavior of the target cells in several ways: (a) directly stimulate the cells by a surface interaction [113]; (b) transfer receptors from the cell of origin to the target cell [114]; (c) deliver proteins to target cells [115]; (d) mediate a horizontal transfer of mRNA and microRNA inducing epigenetic changes in the target cell [116–118]. Therefore, understanding the modulation of MVs' therapeutic effect upon AKI may provide insight into the molecular mechanisms. For example, MVs expressing ICAM1 at their surface can interact with the lymphocyte function-associated antigen 1 (LFA1) to activate T cells [119]. MVs expressing the delta-like 4 (Dll4) may activate angiogenesis and axon growth by interacting with Notch receptors expressed by endothelial or nerve cells, respectively [120]. MVs derived from MSCs may activate a proliferative programme in tubular epithelial cells that survived injury both* in vitro* and in a glycerol-induced model of AKI in severe combined immunodeficiency (SCID) mice [121]. Several other studies also reported that MSCs-MVs might favor the kidney repair in nonlethal toxic and ischemic AKI. They were found to exert a prosurvival and antiapoptotic effect on renal tubular cells* in vitro* and* in vivo* [122–124].

## 7. Summary

**Figure 1:**
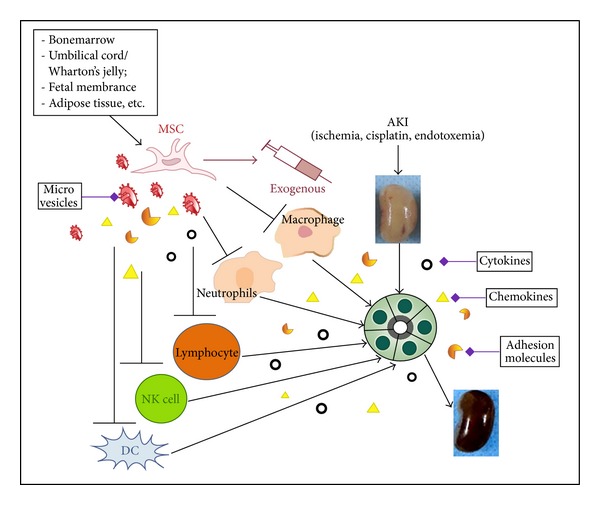
Exogenous MSCs likely involve paracrine effects in regulating the inflammatory mediators to ameliorate AKI. They exert protective and reparative effects in the treatment of AKI by regulating inflammatory mediators like cytokines, chemokines, neutrophils, lymphocytes, NK cells, DCs, and macrophages. MSCs ultimately improve the kidney's structure and function.

Inflammation is now believed to play a major role in the pathophysiology of AKI. Inflammatory mediators are produced to regulate the inflammation in injured kidney. If tubular epithelial and endothelial injury exceeds over the regenerative potential, injured kidney might progress to interstitial fibrosis and even chronic kidney disease. Therefore, controlling kidney inflammation and promoting epithelial/endothelial repair are probably the best ways to target AKI and to maintain renal function. The role of MSCs in cell-based therapies for AKI is under intensive investigation. Preclinical studies indicate that administered MSCs can both ameliorate renal injury and accelerate renal repair. The mechanisms responsible for their protective and regenerative effects are incompletely understood. However, a concept is now clearer that exogenous MSCs likely involve paracrine effects in regulating the inflammatory mediators to ameliorate kidney injury (Figure 1). Some researchers also found that MVs derived from MSCs can alleviate AKI. By regulating inflammatory mediators like cytokines, chemokines, neutrophils, lymphocytes, NK cells, DCs, and macrophages, MSCs could reduce the kidney inflammation, restore the healthy epithelium and endothelium, and ultimately improve the kidney structure and function.

## Figures and Tables

**Table 1:** Preclinical studies using mesenchymal stem cells isolated from various sources to treat acute kidney injury. All the results showed that MSCs could ameliorate the kidney injury [18–28] (main mechanism involving inflammatory mediators).

MSC source	Type of AKI model	Route of MSC delivery	Main mechanism	Reference
Rat BM-MSCs	40 min bilateral IRI	Intra-aortic delivery via left carotid artery	↓IL-1*β*, TNF-*α*, IFN-*γ*, iNOS in kidney	[18]
			↑IL-10, bFGF, TGF-a in kidney	

Human BM-MSCs	Cisplatin-induced kidney injury	i.p. injection	↓MIP-2, KC, CCL-2, IFN-*γ*, IL-6 in serum	[19]
			↑P-Akt in kidney	

Mouse BM-MSCs	Sepsis-associated AKI.	Tail vein	↓IL-17, IL-6, IFN-*γ*, TNF-*α* CXCL1, CXCL2, CXCL5, CCL2, CCL3 in kidney	[20]
			↑IL-10 in kidney	

Mouse AD-MSCs	45 min unilateral IRI	Tail vein	↓CCL3, IL-1b, CCL5, CXCL-10, IL-17 in serum;	[21]
			↓CCL2, CCL3, CCL5, KC in kidney	

Rat fetal membrane MSCs (FM-MSCs)	60 min unilateral IRI	Tail vein	↓infiltration of macrophages and T cells; ↓IL-6 and MCP-1 levels in kidney;	[22]
			↑IL-10 levels in serum	

Rat BM-MSCs	60 min bilateral IRI	i.v. injection	↓IL-1*β*, IL-6, TNF-*α* in kidney	[23]
			↑IL-4 and IL-10 in kidney	

Human umbilical cord-MSCs	60 min bilateral IRI	Intra-aortic delivery via left carotid artery	↓IL-1*β*, IL-6, TNF-*α* in kidney	[24]

Rat BM-MSCs	60 min bilateral IRI	i.v. injection	↓IL-1*β* in kidney	[25]
			↑IL-4 in kidney	

Rat BM-MSCs	Gentamicin-induced kidney injury	i.v. injection	↓IL6, INF-*γ* and TNF-*α* levels in serum	[26]
			↑IL-10 levels in serum	

Human Wharton's jelly-MSCs	45 min unilateral IRI	Tail vein	↑IL-10, heme oxygenase (HO)-1 and HGF in kidney ↑p-Akt in kidney	[27]

Rat AD-MSCs	60 min bilateral IRI	Intrarenal injection and intravenous injection	↓oxidative stress ↓inflammatory response ↓bcl-2, eNOS in kidney	[28]
			↑IL-10, TNF-*α* in kidney	

**Table 2:** Comparative data on chemokines and chemokine receptors expressed in different human MSC populations [50, 53–60].

Human MSCs: tissue sources	Chemokines	Chemokine receptors
Bone marrow-MSCs	CXCL1, CXCL2, CXCL5, CXCL6, CXCL8, CXCL12, CXCL13	CXCR1, CXCR2, CXCR3, CXCR4, CXCR5, CXCR6
	CCL2, CCL3, CCL13, CCL17, CCL18	CCR1, CCR2, CCR3, CCR4, CCR5, CCR6, CCR7, CCR8, CCR9, CCRL1, CCRL2
		CX3CR1

Umbilical cord (Wharton's jelly)-derived MSCs	CXCL1, CXCL2, CXCL5, CXCL6, CXCL8, CXCL12, CXCL13	CXCR3, CXCR5
	CCL2, CCL3, CCL13, CCL17, CCL18	CCR1, CCR3, CCR5, CCR6, CCR7, CCRL1, CCRL2
		CX3CR1

Adipose tissue-MSCs	CXCL1, CXCL2, CXCL5, CXCL6, CXCL8, CXCL16	CXCR2, CXCR4, CXCR5, CXCR6
	CCL2, CCL8,	CCR1, CCR7

## References

[1] Legrand M, Mik EG, Johannes T, Payen D, Ince C (2008). Renal hypoxia and dysoxia after reperfusion of the ischemic kidney. *Molecular Medicine*.

[2] Bellomo R, Wan L, Langenberg C, May C (2008). Septic acute kidney injury: new concepts. *Nephron: Experimental Nephrology*.

[3] Pannu N, Nadim MK (2008). An overview of drug-induced acute kidney injury. *Critical Care Medicine*.

[4] Bonventre JV, Yang L (2011). Cellular pathophysiology of ischemic acute kidney injury. *The Journal of Clinical Investigation*.

[5] Bonventre JV, Zuk A (2004). Ischemic acute renal failure: an inflammatory disease?. *Kidney International*.

[6] Friedewald JJ, Rabb H (2004). Inflammatory cells in ischemic acute renal failure. *Kidney International*.

[7] Umehara H, Goda S, Imai T (2001). Fractalkine, a CX_3_C-chemokine, functions predominantly as an adhesion molecule in monocytic cell line THP-1. *Immunology and Cell Biology*.

[8] Rodríguez-Iturbe B, García GG (2010). The role of tubulointerstitial inflammation in the progression of chronic renal failure. *Nephron: Clinical Practice*.

[9] Yalavanthy R, Edelstein CL (2008). Therapeutic and predictive targets of AKI. *Clinical Nephrology*.

[10] Malgieri A, Kantzari E, Patrizi MP, Gambardella S (2010). Bone marrow and umbilical cord blood human mesenchymal stem cells: state of the art. *International Journal of Clinical and Experimental Medicine*.

[11] Gao J, Liu R, Wu J (2012). The use of chitosan based hydrogel for enhancing the therapeutic benefits of adipose-derived MSCs for acute kidney injury. *Biomaterials*.

[12] Zhu SF, Zhong ZN, Fu XF (2013). Comparison of cell proliferation, apoptosis, cellular morphology and ultrastructure between human umbilical cord and placenta-derived mesenchymal stem cells. *Neuroscience Letters*.

[13] Jones BA, Pei M (2012). Synovium-derived stem cells: a tissue-specific stem cell for cartilage engineering and regeneration. *Tissue Engineering B: Reviews*.

[14] Tamaki T, Soeda S, Hashimoto H (2013). 3D reconstitution of nerve-blood vessel networks using skeletal muscle-derived multipotent stem cell sheet pellets. *Regenerative Medicine*.

[15] Dominici M, le Blanc K, Mueller I (2006). Minimal criteria for defining multipotent mesenchymal stromal cells. The International Society for Cellular Therapy position statement. *Cytotherapy*.

[16] Tögel F, Weiss K, Yang Y, Hu Z, Zhang P, Westenfelder C (2007). Vasculotropic, paracrine actions of infused mesenchymal stem cells are important to the recovery from acute kidney injury. *American Journal of Physiology: Renal Physiology*.

[17] Cheng K, Rai P, Plagov A (2013). Transplantation of bone marrow-derived MSCs improves cisplatinum-induced renal injury through paracrine mechanisms. *Experimental and Molecular Pathology*.

[18] Tögel F, Hu Z, Weiss K, Isaac J, Lange C, Westenfelder C (2005). Administered mesenchymal stem cells protect against ischemic acute renal failure through differentiation-independent mechanisms. *American Journal of Physiology: Renal Physiology*.

[19] Eliopoulos N, Zhao J, Bouchentouf M (2010). Human marrow-derived mesenchymal stromal cells decrease cisplatin renotoxicity *in vitro* and *in vivo* and enhance survival of mice post-intraperitoneal injection. *American Journal of Physiology: Renal Physiology*.

[20] Luo CJ, Zhang FJ, Zhang L (2014). Mesenchymal stem cells ameliorate sepsis-associated acute kidney injury in mice. *Shock*.

[21] Furuichi K, Shintani H, Sakai Y (2012). Effects of adipose-derived mesenchymal cells on ischemia-reperfusion injury in kidney. *Clinical and Experimental Nephrology*.

[22] Tsuda H, Yamahara K, Otani K Transplantation of allogenic fetal membrane-derived mesenchymal stem cells protect against ischemia-reperfusion-induced acute kidney injury.

[23] Semedo P, Palasio CG, Oliveira CD (2009). Early modulation of inflammation by mesenchymal stem cell after acute kidney injury. *International Immunopharmacology*.

[24] Cao HL, Qian H, Xu WR (2010). Mesenchymal stem cells derived from human umbilical cord ameliorate ischemia/reperfusion-induced acute renal failure in rats. *Biotechnology Letters*.

[25] Semedo P, Wang PM, Andreucci TH (2007). Mesenchymal stem cells ameliorate tissue damages triggered by renal ischemia and reperfusion injury. *Transplantation Proceedings*.

[26] Reis LA, Borges FT, Simões MJ (2012). Bone marrow-derived mesenchymal stem cells repaired but did not prevent gentamicin-induced acute kidney injury through paracrine effects in rats. *PLoS ONE*.

[27] Du T, Cheng J, Zhong L (2012). The alleviation of acute and chronic kidney injury by human Wharton's jelly-derived mesenchymal stromal cells triggered by ischemia-reperfusion injury via an endocrine mechanism. *Cytotherapy*.

[28] Chen Y-T, Sun C-K, Lin Y-C (2011). Adipose-derived mesenchymal stem cell protects kidneys against ischemia-reperfusion injury through suppressing oxidative stress and inflammatory reaction. *Journal of Translational Medicine*.

[29] Karshovska E, Weber C, von Hundelshausen P (2013). Platelet chemokines in health and disease. *Thrombosis and Haemostasis*.

[30] Imaizumi T, Aizawa-Yashiro T, Tsuruga K (2012). Melanoma differentiation-associated gene 5 regulates the expression of a chemokine CXCL10 in human mesangial cells: implications for chronic inflammatory renal diseases. *The Tohoku Journal of Experimental Medicine*.

[31] Furuichi K, Wada T, Yokoyama H, Kobayashi K-I (2002). Role of cytokines and chemokines in renal ischemia-reperfusion injury. *Drug News & Perspectives*.

[32] Akcay A, Nguyen Q, Edelstein CL (2009). Mediators of inflammation in acute kidney injury. *Mediators of Inflammation*.

[33] Patel J, Pancholi N, Gudehithlu KP (2012). Stem cells from foreign body granulation tissue accelerate recovery from acute kidney injury. *Nephrology Dialysis Transplantation*.

[34] Faubel S, Lewis EC, Reznikov L (2007). Cisplatin-induced acute renal failure is associated with an increase in the cytokines interleukin (IL)-1*β*, IL-18, IL-6, and neutrophil infiltration in the kidney. *The Journal of Pharmacology and Experimental Therapeutics*.

[35] Zhang B, Ramesh G, Uematsu S, Akira S, Reeves WB (2008). TLR4 signaling mediates inflammation and tissue injury in nephrotoxicity. *Journal of the American Society of Nephrology*.

[36] Ramesh G, Reeves WB (2003). TNFR2-mediated apoptosis and necrosis in cisplatin-induced acute renal failure. *American Journal of Physiology: Renal Physiology*.

[37] Goes N, Urmson J, Ramassar V, Halloran PF (1995). Ischemic acute tubular necrosis induces an extensive local cytokine response: evidence for induction of interferon-*γ*, transforming growth factor-*β*1, granulocyte-macrophage colony-stimulating factor, interleukin-2, and interleukin-10. *Transplantation*.

[38] Thurman JM, Lenderink AM, Royer PA (2007). C3a is required for the production of CXC chemokines by tubular epithelial cells after renal ishemia/reperfusion. *The Journal of Immunology*.

[39] Rice JC, Spence JS, Yetman DL, Safirstein RL (2002). Monocyte chemoattractant protein-1 expression correlates with monocyte infiltration in the post-ischemic kidney. *Renal Failure*.

[40] Zhao JJ, Liu JL, Liu L, Jia HY (2014). Protection of mesenchymal stem cells on acute kidney injury. *Molecular Medicine Reports*.

[41] Bi B, Schmitt R, Israilova M, Nishio H, Cantley LG (2007). Stromal cells protect against acute tubular injury via an endocrine effect. *Journal of the American Society of Nephrology*.

[42] Imberti B, Morigi M, Tomasoni S (2007). Insulin-like growth factor-1 sustains stem cell-mediated renal repair. *Journal of the American Society of Nephrology*.

[43] Uccelli A, Pistoia V, Moretta L (2007). Mesenchymal stem cells: a new strategy for immunosuppression?. *Trends in Immunology*.

[44] Oskowitz A, McFerrin H, Gutschow M, Carter ML, Pochampally R (2011). Serum-deprived human multipotent mesenchymal stromal cells (MSCs) are highly angiogenic. *Stem Cell Research*.

[45] Qi S, Wu D (2013). Bone marrow-derived mesenchymal stem cells protect against cisplatin-induced acute kidney injury in rats by inhibiting cell apoptosis. *International Journal of Molecular Medicine*.

[46] Segerer S, Nelson PJ, Schlondorff D (2000). Chemokines, chemokine receptors, and renal disease: from basic science to pathophysiologic and therapeutic studies. *Journal of the American Society of Nephrology*.

[47] Furuichi K, Kaneko S, Wada T (2009). Chemokine/chemokine receptor-mediated inflammation regulates pathologic changes from acute kidney injury to chronic kidney disease. *Clinical and Experimental Nephrology*.

[48] Togel F, Isaac J, Hu ZM, Weiss K, Westenfelder C (2005). Renal SDF-1 signals mobilization and homing of CXCR4-positive cells to the kidney after ischemic injury. *Kidney International*.

[49] Furuichi K, Wada T, Iwata Y (2002). Administration of FR167653, a new anti-inflammatory compound, prevents renal ischaemia/reperfusion injury in mice. *Nephrology Dialysis Transplantation*.

[50] Balasubramanian S, Venugopal P, Sundarraj S, Zakaria Z, Majumdar AS, Ta M (2012). Comparison of chemokine and receptor gene expression between Wharton’s jelly and bone marrow-derived mesenchymal stromal cells. *Cytotherapy*.

[51] Hung S-C, Pochampally RR, Hsu S-C (2007). Short-term exposure of multipotent stromal cells to low oxygen increases their expression of CX3CR1 and CXCR4 and their engraftment *in vivo*. *PLoS ONE*.

[52] Liu H, Liu S, Li Y (2012). The role of SDF-1-CXCR4/CXCR7 axis in the therapeutic effects of hypoxia-preconditioned mesenchymal stem cells for renal ischemia/ reperfusion injury. *PLoS ONE*.

[53] Honczarenko M, Le Y, Swierkowski M, Ghiran I, Glodek AM, Silberstein LE (2006). Human bone marrow stromal cells express a distinct set of biologically functional chemokine receptors. *Stem Cells*.

[54] Kim DS, Lee MW, Yoo KH (2014). Gene expression profiles of human adipose tissue-derived mesenchymal stem cells are modified by cell culture density. *PLoS ONE*.

[55] Baek SJ, Kang SK, Ra JC (2011). *In vitro* migration capacity of human adipose tissue-derived mesenchymal stem cells reflects their expression of receptors for chemokines and growth factors. *Experimental & Molecular Medicine*.

[56] Sordi V, Malosio ML, Marchesi F (2005). Bone marrow mesenchymal stem cells express a restricted set of functionally active chemokine receptors capable of promoting migration to pancreatic islets. *Blood*.

[57] Ponte AL, Marais E, Gallay N (2007). The *in vitro* migration capacity of human bone marrow mesenchymal stem cells: comparison of chemokine and growth factor chemotactic activities. *Stem Cells*.

[58] Ringe J, Strassburg S, Neumann K (2007). Towards *in situ* tissue repair: human mesenchymal stem cells express chemokine receptors CXCR1, CXCR2 and CCR2, and migrate upon stimulation with CXCL8 but not CCL2. *Journal of Cellular Biochemistry*.

[59] Zhang H, Ning H, Banie L (2010). Adipose tissue-derived stem cells secrete CXCL5 cytokine with chemoattractant and angiogenic properties. *Biochemical and Biophysical Research Communications*.

[60] Fox JM, Chamberlain G, Ashton BA, Middleton J (2007). Recent advances into the understanding of mesenchymal stem cell trafficking. *British Journal of Haematology*.

[61] Albelda SM, Smith CW, Ward PA (1994). Adhesion molecules and inflammatory injury. *The FASEB Journal*.

[62] Molitoris BA, Marrs J (1999). The role of cell adhesion molecules in ischemic acute renal failure. *American Journal of Medicine*.

[63] Kelly KJ, Williams WW, Colvin RB, Bonventre JV (1994). Antibody to intercellular adhesion molecule 1 protects the kidney against ischemic injury. *Proceedings of the National Academy of Sciences of the United States of America*.

[64] Kelly KJ, Williams WW, Colvin RB (1996). Intercellular adhesion molecule-1-deficient mice are protected against ischemic renal injury. *The Journal of Clinical Investigation*.

[65] Hara Y, Stolk M, Ringe J (2011). *In vivo* effect of bone marrow-derived mesenchymal stem cells in a rat kidney transplantation model with prolonged cold ischemia. *Transplant International*.

[66] Hagiwara M, Shen B, Chao L, Chao J (2008). Kallikrein-modified mesenchymal stem cell implantation provides enhanced protection against acute ischemic kidney injury by inhibiting apoptosis and inflammation. *Human Gene Therapy*.

[67] Dragun D, Hoff U, Park JK (2000). Ischemia-reperfusion injury in renal transplantation is independent of the immunologic background. *Kidney International*.

[68] Rüster B, Göttig S, Ludwig RJ (2006). Mesenchymal stem cells display coordinated rolling and adhesion behavior on endothelial cells. *Blood*.

[69] de Filippo K, Dudeck A, Hasenberg M (2013). Mast cell and macrophage chemokines CXCL1/CXCL2 control the early stage of neutrophil recruitment during tissue inflammation. *Blood*.

[70] Yagi H, Soto-Gutierrez A, Navarro-Alvarez N (2010). Reactive bone marrow stromal cells attenuate systemic inflammation via sTNFR1. *Molecular Therapy*.

[71] Morigi M, Rota C, Montemurro T (2010). Life-sparing effect of human cord blood-mesenchymal stem cells in experimental acute kidney injury. *Stem Cells*.

[72] Wang Q, Zhu H, Zhou WG (2013). N-acetylcysteine- pretreated human embryonic mesenchymal stem cell administration protects against bleomycin-induced lung injury. *The American Journal of the Medical Sciences*.

[73] Rabb H (2002). The T cell as a bridge between innate and adaptive immune systems: implications for the kidney. *Kidney International*.

[74] Hu J, Zhang L, Wang N (2013). Mesenchymal stem cells attenuate ischemic acute kidney injury by inducing regulatory T cells through splenocyte interactions. *Kidney International*.

[75] Kim M-G, Lee SY, Ko YS (2011). CD4^+^ CD25^+^ regulatory T cells partially mediate the beneficial effects of FTY720, a sphingosine-1-phosphate analogue, during ischaemia/reperfusion-induced acute kidney injury. *Nephrology Dialysis Transplantation*.

[76] Wang YM, Zhang GY, Hu M (2012). CD8^+^ regulatory T cells induced by T cell vaccination protect against autoimmune nephritis. *Journal of the American Society of Nephrology*.

[77] Ye Z, Wang Y, Xie H-Y, Zheng S-S (2008). Immunosuppressive effects of rat mesenchymal stem cells: involvement of CD4^+^CD25^+^ regulatory T cells. *Hepatobiliary & Pancreatic Diseases International*.

[78] Kim J-A, Hong S, Lee B (2007). The inhibition of T-cells proliferation by mouse mesenchymal stem cells through the induction of p16^INK4A^-cyclin D1/cdk4 and p21^waf1^, p27^kip1^-cyclin E/cdk2 pathways. *Cellular Immunology*.

[79] Nauta AJ, Fibbe WE (2007). Immunomodulatory properties of mesenchymal stromal cells. *Blood*.

[80] Duffy MM, Pindjakova J, Hanley SA (2011). Mesenchymal stem cell inhibition of T-helper 17 cell- differentiation is triggered by cell-cell contact and mediated by prostaglandin E2 via the EP4 receptor. *European Journal of Immunology*.

[81] Yañez R, Oviedo A, Aldea M, Bueren JA, Lamana ML (2010). Prostaglandin E2 plays a key role in the immunosuppressive properties of adipose and bone marrow tissue-derived mesenchymal stromal cells. *Experimental Cell Research*.

[82] Najar M, Raicevic G, Boufker HI (2010). Mesenchymal stromal cells use PGE2 to modulate activation and proliferation of lymphocyte subsets: combined comparison of adipose tissue, Wharton’s jelly and bone marrow sources. *Cellular Immunology*.

[83] Sakaguchi S, Yamaguchi T, Nomura T, Ono M (2008). Regulatory T cells and immune tolerance. *Cell*.

[84] Burne-Taney MJ, Ascon DB, Daniels F, Racusen L, Baldwin W, Rabb H (2003). B cell deficiency confers protection from renal ischemia reperfusion injury. *The Journal of Immunology*.

[85] Schena F, Gambini C, Gregorio A (2010). Interferon-*γ*-dependent inhibition of B cell activation by bone marrow-derived mesenchymal stem cells in a murine model of systemic lupus erythematosus. *Arthritis & Rheumatism*.

[86] Ma X, Che N, Gu Z (2012). Allogenic mesenchymal stem cell transplantation ameliorates nephritis in lupus mice via inhibition of B-cell activation. *Cell Transplantation*.

[87] Cerwenka A, Lanier LL (2001). Natural killer cells, viruses and cancer. *Nature Reviews Immunology*.

[88] Zhang Z-X, Wang S, Huang X (2008). NK cells induce apoptosis in tubular epithelial cells and contribute to renal ischemia-reperfusion injury. *The Journal of Immunology*.

[89] Pradier A, Passweg J, Villard J, Kindler V (2011). Human bone marrow stromal cells and skin fibroblasts inhibit natural killer cell proliferation and cytotoxic activity. *Cell Transplantation*.

[90] Selmani Z, Naji A, Zidi I (2008). Human leukocyte antigen-G5 secretion by human mesenchymal stem cells is required to suppress T lymphocyte and natural killer function and to induce CD4^+^CD25^high^FOXP3^+^ regulatory T cells. *Stem Cells*.

[91] Ribeiro A, Laranjeira P, Mendes S (2013). Mesenchymal stem cells from umbilical cord matrix, adipose tissue and bone marrow exhibit different capability to suppress peripheral blood B, natural killer and T cells. *Stem Cell Research & Therapy*.

[92] Zhao ZG, Cao Z, Xu W (2012). Immune protection function of multipotent mesenchymal stromal cells: role of transforming growth factor-*β*1. *Cancer Investigation*.

[93] Nikolic-Paterson DJ, Atkins RC (2001). The role of macrophages in glomerulonephritis. *Nephrology Dialysis Transplantation*.

[94] Cao Q, Zheng D, Wang YP, Harris DC (2011). Macrophages and dendritic cells for treating kidney disease. *Nephron: Experimental Nephrology*.

[95] Lu H, Huang D, Saederup N, Charo IF, Ransohoff RM, Zhou L (2011). Macrophages recruited via CCR2 produce insulin-like growth factor-1 to repair acute skeletal muscle injury. *The FASEB Journal*.

[96] Mosser DM, Edwards JP (2008). Exploring the full spectrum of macrophage activation. *Nature Reviews Immunology*.

[97] Ricardo SD, van Goor H, Eddy AA (2008). Macrophage diversity in renal injury and repair. *The Journal of Clinical Investigation*.

[98] Anders H-J, Ryu M (2011). Renal microenvironments and macrophage phenotypes determine progression or resolution of renal inflammation and fibrosis. *Kidney International*.

[99] Luo J, Zhao X, Tan Z, Su Z, Meng F, Zhang M (2013). Mesenchymal-like progenitors derived from human embryonic stem cells promote recovery from acute kidney injury via paracrine actions. *Cytotherapy*.

[100] Lee S, Huen S, Nishio H (2011). Distinct macrophage phenotypes contribute to kidney injury and repair. *Journal of the American Society of Nephrology*.

[101] Li W, Zhang Q, Wang M (2013). Macrophages are involved in the protective role of human umbilical cord-derived stromal cells in renal ischemia-reperfusion injury. *Stem Cell Research*.

[102] Okusa MD, Li L (2012). Dendritic cells in acute kidney injury: cues from the microenvironment. *Transactions of the American Clinical and Climatological Association*.

[103] Kim MG, Kim SH, Noh H (2013). CD11c^+^ cells partially mediate the renoprotective effect induced by bone marrow-derived mesenchymal stem cells. *PLoS ONE*.

[104] Zhang Y, Cai W, Huang Q (2014). Mesenchymal stem cells alleviate bacteria-induced liver injury in mice by inducing regulatory dendritic cells. *Hepatology*.

[105] Han Z, Jing Y, Zhang S (2012). The role of immunosuppression of mesenchymal stem cells in tissue repair and tumor growth. *Cell & Bioscience*.

[106] Tian H, Lu Y, Shah SP, Wang Q, Hong S (2012). 14S,21*R*-dihydroxy-docosahexaenoic acid treatment enhances mesenchymal stem cell amelioration of renal ischemia/reperfusion injury. *Stem Cells and Development*.

[107] Kronsteiner B, Peterbauer-Scherb A, Grillari-Voglauer R (2011). Human mesenchymal stem cells and renal tubular epithelial cells differentially influence monocyte-derived dendritic cell differentiation and maturation. *Cellular Immunology*.

[108] Spaggiari GM, Abdelrazik H, Becchetti F, Moretta L (2009). MSCs inhibit monocyte-derived DC maturation and function by selectively interfering with the generation of immature DCs: central role of MSC-derived prostaglandin E2. *Blood*.

[109] Ratajczak J, Miekus K, Kucia M (2006). Embryonic stem cell-derived microvesicles reprogram hematopoietic progenitors: evidence for horizontal transfer of mRNA and protein delivery. *Leukemia*.

[110] Camussi G, Deregibus MC, Bruno S, Cantaluppi V, Biancone L (2010). Exosomes/microvesicles as a mechanism of cell-to-cell communication. *Kidney International*.

[111] Camussi G, Deregibus MC, Cantaluppi V (2013). Role of stem-cell-derived microvesicles in the paracrine action of stem cells. *Biochemical Society Transactions*.

[112] Wu S, Ju GQ, Du T (2013). Microvesicles derived from human umbilical cord Wharton's jelly mesenchymal stem cells attenuate bladder tumor cell growth *in vitro* and *in vivo*. *PLoS ONE*.

[113] Polgar J, Matuskova J, Wagner DD (2005). The P-selectin, tissue factor, coagulation triad. *Journal of Thrombosis and Haemostasis*.

[114] Kim JW, Wieckowski E, Taylor DD, Reichert TE, Watkins S, Whiteside TL (2005). Fas ligand-positive membranous vesicles isolated from sera of patients with oral cancer induce apoptosis of activated T lymphocytes. *Clinical Cancer Research*.

[115] Ratajczak J, Wysoczynski M, Hayek F, Janowska-Wieczorek A, Ratajczak MZ (2006). Membrane-derived microvesicles: important and underappreciated mediators of cell-to-cell communication. *Leukemia*.

[116] Collino F, Deregibus MC, Bruno S (2010). Microvesicles derived from adult human bone marrow and tissue specific mesenchymal stem cells shuttle selected pattern of miRNAs. *PLoS ONE*.

[117] Valadi H, Ekstrom K, Bossios A, Sjöstrand M, Lee JJ, Lötvall JO (2007). Exosome-mediated transfer of mRNAs and microRNAs is a novel mechanism of genetic exchange between cells. *Nature Cell Biology*.

[118] Ramachandran S, Palanisamy V (2012). Horizontal transfer of RNAs: exosomes as mediators of intercellular communication. *Wiley Interdisciplinary Reviews: RNA*.

[119] Nolte-’t Hoen EN, Buschow SI, Anderton SM, Stoorvogel W, Wauben MHM (2009). Activated T cells recruit exosomes secreted by dendritic cells via LFA-1. *Blood*.

[120] Sheldon H, Heikamp E, Turley H (2010). New mechanism for Notch signaling to endothelium at a distance by Delta-like 4 incorporation into exosomes. *Blood*.

[121] Bruno S, Grange C, Deregibus MC (2009). Mesenchymal stem cell-derived microvesicles protect against acute tubular injury. *Journal of the American Society of Nephrology*.

[122] Bruno S, Grange C, Collino F (2012). Microvesicles derived from mesenchymal stem cells enhance survival in a lethal model of acute kidney injury. *PLoS ONE*.

[123] Zhou Y, Xu H, Xu W (2013). Exosomes released by human umbilical cord mesenchymal stem cells protect against cisplatin-induced renal oxidative stress and apoptosis *in vivo* and *in vitro*. *Stem Cell Research & Therapy*.

[124] Gatti S, Bruno S, Deregibus MC (2011). Microvesicles derived from human adult mesenchymal stem cells protect against ischaemia-reperfusion-induced acute and chronic kidney injury. *Nephrology Dialysis Transplantation*.

